# The Device of Ethylene Vinyl Acetate Sheet for Trismus Caused by Bilateral Mandible Fractures

**DOI:** 10.1155/2021/8340485

**Published:** 2021-08-25

**Authors:** Yoshiaki Ihara, Yuka Nakamichi, Yuichi Tashimo, Shinji Nozue, Kota Hayashi, Mitsunori Ishiguro, Koji Takahashi

**Affiliations:** Division of Oral Rehabilitation Medicine, Department of Special Needs Dentistry, School of Dentistry, Showa University, Tokyo, Japan

## Abstract

Trismus is commonly caused by temporomandibular joint disorders and maxillofacial fractures. We report the case of a 62-year-old woman with trismus and difficulty in mastication caused by bilateral mandibular fractures. She had a maximal interincisal opening distance (MID) of 22 mm. Mouth-opening training was administered using a novel dental mouth-training device custom-made using ethylene vinyl acetate sheets and according to the dentition and extent of mouth-opening of the patient. After 2 months of training, the MID increased to 42 mm. With adequate training, this device is effective in treating trismus due to scarring.

## 1. Introduction

Trismus or restricted mouth-opening is commonly caused by temporomandibular joint (TMJ) disorders, fractures in the maxillofacial region, and treatment of head and neck cancers. The maximal interincisal opening distance (MID), the distance between the upper and lower incisors at maximum opening, is usually measured to assess mouth-opening. Trismus has been previously diagnosed with MID values of 30 mm [1, 2], 35 mm [3, 4], and 40 mm [5, 6]. Traumatic facial fractures often result in malocclusion after treatment due to impaired opening or deviation of the occlusal position. Although manual methods and off-the-shelf mouth-training devices have been used in the past for the treatment of trismus, it is often difficult to achieve sustained therapeutic effects or ensure that the patient can train themselves at home. It was reported that approximately one in four patients does not adhere to the prescribed exercise therapy [7]. We fabricated a novel device for mouth-opening exercises using a dental thermoplastic sheet, which can be fitted according to the individual's dentition and mouth-opening. Herein, we report the application of this device in a patient with trismus following bilateral mandible fractures, which resulted in good therapeutic outcomes.

## 2. Case Report

**Figure 1:**
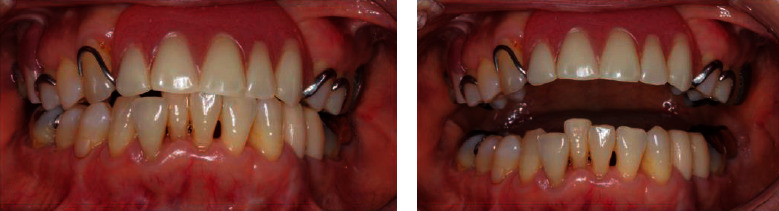
Preoperative views: (a) front view and (b) maximum mandibular opening at preoperation.

**Figure 2:**
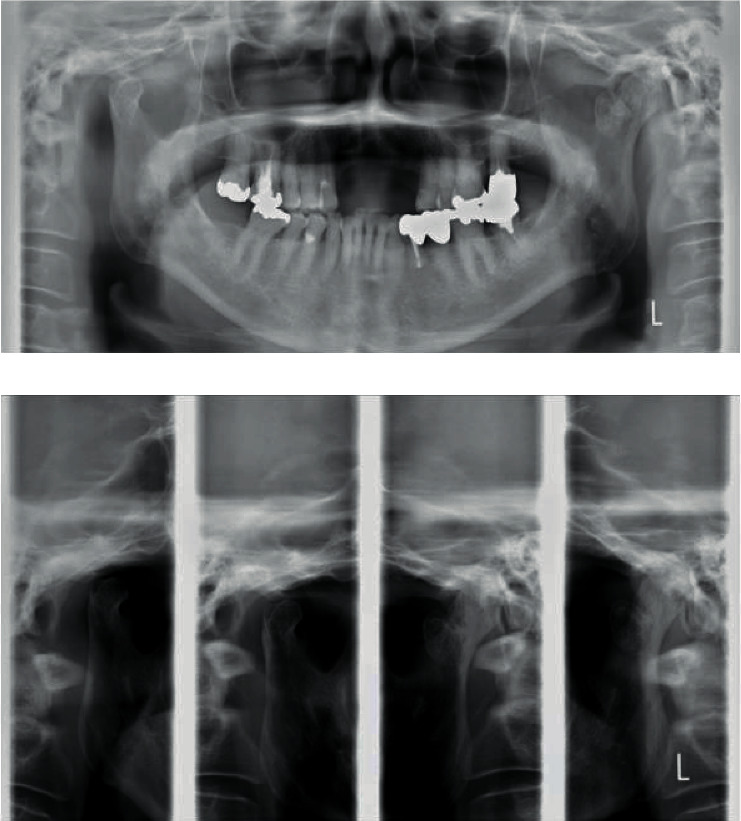
Radiographic findings: (a) panoramic radiograph and (b) lateral projection of the temporomandibular joint.

**Figure 3:**
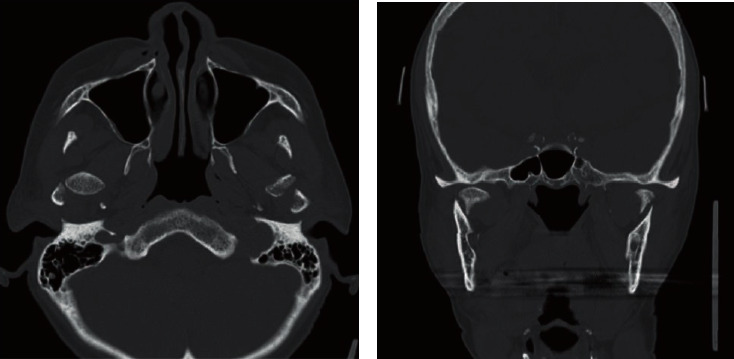
CT findings: (a) sagittal reconstruction of CT images and (b) coronal reconstruction of CT images. The bilateral condylar fractures were observed.

A 62-year-old woman complained of trismus and difficulty in mastication following bilateral mandibular fractures. Six months prior, she had lost consciousness in a bus and hit her head when she fell, which resulted in fractures of the jawbone and anterior part of the maxilla, among other injuries. Subsequently, she was immobilized for 3 months. She was referred to our department because of persistent pain and no improvement in mouth-opening despite rehabilitation with a wooden device. She complained of pain in the molar during mouth-opening exercise, which reflected poor adherence to mouth-opening training. She lost her maxillary incisors and used a partial denture. Thus, we measured her MID between incisors of a partial denture and lower incisors. The MID was 22 mm, and she experienced pain on attempting to open the mouth further (Figure 1). Radiography and CT images revealed displacement of the left and right temporomandibular articular heads due to fracture (Figures 2 and 3). Tooth mobility was noted in the maxillary annular tooth. The General Oral Health Assessment Index (GOHAI) [8] score was 29 points at the first visit. Regarding feeding function, the Fujishima's Food Intake Level Scale [9] value was level 8 (the patient ate three meals, avoiding food that is particularly difficult to swallow).

**Figure 4:**
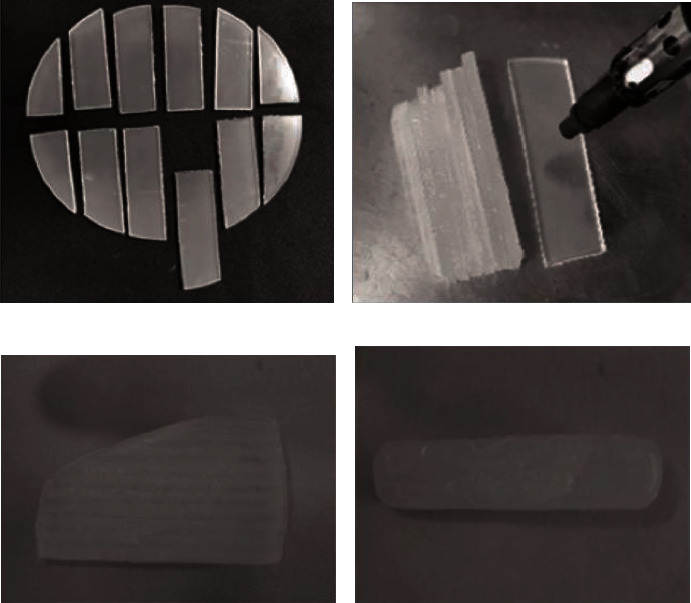
Fabrication of the device. (a) A 4 mm thick ethylene vinyl acetate (EVA) sheet was cut into 2 × 6 cm pieces. (b) The surface of the sheets was softened using a burner, and blocks were created. (c) Lateral view of the device. (d) Top view of the device.

**Figure 5:**
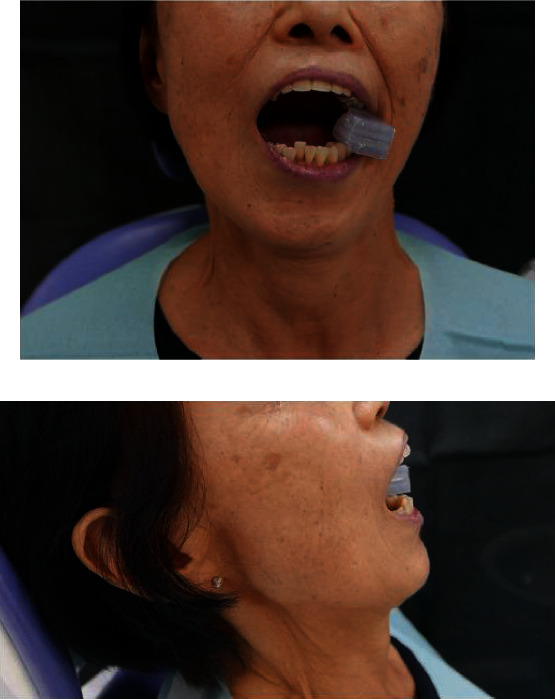
Mouth-opening training with the device. (a, b) The patient received instructions on how to insert the device and advance it in the direction of the molars.

The patient was treated with intra- and extraoral massaging and a home exercise program to increase the MID using a device made of ethylene vinyl acetate (EVA). A 4 mm thick EVA sheet was cut into 2 × 6 mm pieces. The surface of the sheets was then softened using a burner, and blocks were created by stacking the sheets (Figure 4). The thickness of the block was adjusted according to the MID. Furthermore, the device was slanted and inserted from the thin side. The initial thickness of the device was also decided based on the MID. In our patient with MID of 22 mm, the initial thickness of the thin side was 20 mm. The patient received instructions regarding inserting the device, advancing it in the direction of the molar teeth to increase the mouth-opening, and avoiding overloading the teeth and crest (Figure 5). Each training session of 15 minutes was prescribed twice a day whenever possible. Once the device could be easily inserted, the thickness of the device was increased accordingly.

**Figure 6:**
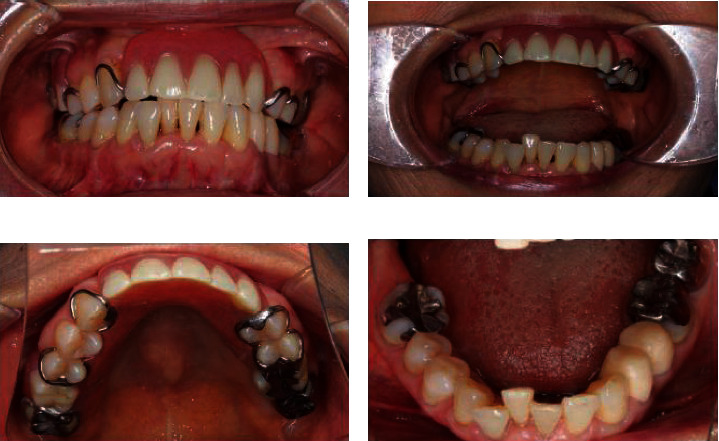
Postoperative views: (a) front view, (b) maximum mandibular opening at postoperation, (c) occlusal view of maxilla, and (d) occlusal view of mandible.

One week after the first visit, the MID improved to 26 mm with training. The thickness of the device was increased to 24 mm to facilitate further mouth-opening since the patient did not complain of any TMJ, muscular, or teeth-related symptoms. After 2 weeks, the MID improved to 32 mm. Tenderness was observed around the left zygomatic area after training, and she was instructed to massage the surrounding area and prescribed exercise therapy for TMJ after mouth-opening training. The patient complained that the increase in the extent of mouth-opening persisted only for approximately 2 hours after the training. Therefore, the patient was instructed to perform mouth-opening training twice a day (morning and evening). After 2 months, the MID before the training session was 35 mm and improved to 42 mm after training (Table 1 and Figure 6). At this stage, the patient was comfortable and did not experience any issues related to trismus in her daily life. Therefore, it was decided to retain the device thickness at the same level to maintain the improvement in mouth-opening. After 12 months, the MID before training was 41 mm. Oral-related quality of life was assessed using GOHAI score, which improved to 34 after 3 months and 40 after 12 months (Table 1). Regarding feeding function, the Fujishima's Food Intake Level Scale value improved to level 10 (normal; there is no dietary restriction, and the patient ingests three meals orally) after 2 months of training and was sustained after 12 months of training (Table 1).

## 3. Discussion

The purpose of this case report was to highlight the use of an EVA device in mouth-opening exercises. The low-load and prolonged-stretch properties of EVA have been reported to be effective in treating the range of motion (ROM) in some pathologies [10]. In this case, a device made of EVA was effective in increasing ROM in the TMJ.

In the present case, more than 6 months had passed since the bilateral mandibular fractures were sustained before the initial visit to our department, which would have resulted in advanced scarring and restricted mouth-opening. It is reported that the mechanism of scar formation in muscle tissues after trauma involves proliferation of mesenchymal tissue due to necrosis, contusions, and hemorrhage caused by trauma [11]. In this case, scarring of the TMJ and the lip area was observed at the initial visit, which might have caused trismus and difficulty in sealing the lips.

Mouth-opening training using a device is effective for trismus caused by scarring [11]. In a previous study [12], TheraBite® (Atos Medical AB; Hörby, Sweden), a wooden mouth-opening device, was used for such training, which was inserted into the patient's dentition and loaded to force mouth-opening. Some studies have reported that mouth-opening training should be implemented for at least 3 months to be effective [13, 14]. In this case, the patient achieved sufficient mouth-opening (42 mm) after 2 months of training, which suggests that the device used in our case is probably more effective than previously reported training devices, such as wooden devices.

The main difference between the older devices and the present device is the training methodology. Previously, devices were used for a shorter duration of training, lasting only few minutes [15]. In contrast, the device in this study was applied for as long as 15 minutes and demonstrated quicker results. In the field of physical therapy, sustained stretching exercises are often used to prevent restriction of ROM in joints [16]. Mouth-opening training uses continuous stretching movements around the TMJ. It is reported that 30 minutes of sustained extension is most effective for sustained stretching exercises [16]. In this case, we initially instructed the patient to use the device for 30 minutes daily. However, it was difficult for the patient to perform 30 consecutive minutes of training. Therefore, we instructed the patient to use it for 15 minutes twice a day. To obtain more effective outcomes, further studies are needed to examine the ideal duration of training.

EVA sheets are commonly used in devices such as mouth guards to prevent trauma during sports. Therefore, such sheets might have little effect on the patient's teeth during mouth-opening training. In this case, the patient's adherence for training with a wooden device was low. However, her adherence improved for training with the current device. One of the possible reasons might be the difference in the material. The wooden device was so hard that the patient often felt pain during training. A previous study reported that most patients felt pain during training and that the pain could affect adherence to training [17]. However, we used EVA sheets for a different purpose, which ensured a sustained load on the teeth at the insertion site during extended periods of training. Further studies involving more patients are necessary to investigate the benefits of this device and its effects on the teeth.

## 4. Conclusion

We effectively used a new device made of EVA in mouth-opening training in a patient with trismus due to bilateral mandibular fractures. The main advantage of this device is that it is not very hard, resulting in less stress on the teeth and less pain during training. Consequently, good adherence to training was achieved to improve the extent of mouth-opening. The oral function-related quality of life also improved with increase in the extent of mouth-opening.

## Figures and Tables

**Table 1:** Measurements before and after mouth-opening training.

	Initial visit	After 2 months	After 12 months
Maximal interincisal opening distance	22 mm	35 mm	41 mm
General Oral Health Assessment Index	29	34	40
Fujishima's Food Intake Level Scale	8	10	10
